# Snake Bite—Rapid Cure by Carbolic Acid

**Published:** 1883-01

**Authors:** 


Snake Bite—Rapid Cure by Carbolic Acid.—Dr. Sereins relates a case of snake bite treated successfully by hypodermic injections of carbolic acid. The patient, a charwoman, 40 years of age, was bitten by a venomous snake on the left foot just below the external malleolus. Half an hour afterward she experienced an intense smarting at the point of the injury and a sensation of constriction in the abdomen and epigastric region. Soon she began to throw off quantities of glairy mucus and bile.

The vomiting was almost incessant, and each attack was preceded by a painful aura starting from the wound, passing up the limb, and radiating toward the stomach. A tourniquet was applied to the limb, and the wound covered with a compress dipped in a solution of ammonia. Dr. Sereins arrived two hours after the woman had been bitten, and found her vomiting and suffering from a sense of impending suffocation. The skin was cold and covered with perspiration, the pulse feeble and beating 110 to the minute; on the external surface of the foot, just below the malleolus, were two little red points, and above them a small blister caused by the ammonia.

The lower part of the leg was enormously swollen, the skin marbled, with here and there yellowish spots and points of ecchymosis, surrounded by small vesicles. The patient complained bitterly of cold. Four hypodermics of a solution of carbolic acid in glycerine (two in fifteen) were given, one in the neighborhood of the bite and three at the upper edge of the œdematous part of the leg. The wound was also bathed with the same solution. In one hour there was a very appreciable reduction of the swelling, the vomiting being less persistent and ceasing entirely in four hours. The next day the constricting ligature was removed from the leg. On the second day the patient had entirely recovered; there was but slight swelling of the leg, and the yellow spots and ecchymosis had disappeared. The case seems remarkable in the rapidity of the cure effected.

Few accidents of this kind are ever recovered from (if at all) short of a fortnight. Then, the woman was not seen until two hours had elapsed after the wound was inflicted. The nervous centres were profoundly affected, as evidenced by the symptoms. Thus the carbolic acid not only destroyed the venom at the point of introduction, but even neutralized its effects upon the general system.—*L'Union Medicale.*

---

Refilling of Prescriptions.—In Wisconsin, any druggist, apothecary, or vendor of medicine is liable to a fine of ten dollars and costs for every time he is convicted of refilling a prescription marked "No Duplicate."

